# Metabolome and transcriptome profiling provide insights into green apple peel reveals light- and UV-B-responsive pathway in anthocyanins accumulation

**DOI:** 10.1186/s12870-021-03121-3

**Published:** 2021-07-24

**Authors:** Ruirui Ding, Xingkai Che, Zhen Shen, Yuanhu Zhang

**Affiliations:** State Key Laboratory of Crop Biology, College of Life Sciences, Shandong Agricultural University, Taian, 271018 China

**Keywords:** Apple, Flavonoids and anthocyanins accumulation, Visible light, UV-B, Metabolomic and transcriptome

## Abstract

**Background:**

In nature, green apple are associated with the accumulation of chlorophyll, while red apple varieties are associated with anthocyanins accumulation. Notably, in this study, the green skin color apple variety ‘white winter pearmain’ treated with ultraviolet-B (UV-B) exhibited red skins and marked anthocyanin accumulation, while visible light could not. But there are few reports on the biosynthesis difference of anthocyanins in green apple by visible light and UV-B-treatment. Here, we explored the difference of metabolites and genes expression level in green apple by transcriptomic and metabolic.

**Results:**

The metabolic analysis revealed that there were 152 and 178 significantly changed metabolites in the visible light and UV-B-treated green apple, respectively, compared to the control, and flavone, flavonol, and anthocyanin were the most significantly increased; and transcriptomic analysis showed that 37,110 and 37,709 differentially expressed genes, including 382 and 475 transcription factors (TFs) were detected in light and UV-B-treatment fruit, respectively. Quantitative reverse transcription PCR (qRT-PCR) results confirmed changes in the expression levels of genes encoding metabolites involved in the flavonoid synthesis pathways. The flavonoid metabolic flux in the UV-B treatment increased the accumulation of cyanidin 3-glucoside and cyanidin 3, 5-diglucoside compared to under the light-treatment. Furthermore, we performed qRT-PCR analysis of anthocyanin biosynthesis genes and predicted the gene of *MD00G1134400* (a UDP glucose-flavonoid 3–0-glucosyltransferase) may be a candidate gene for anthocyanins accumulation and highly expressed in UV-B-treatment fruit. Expression profiles of several transcription factors of the families MYB, bHLH, NAC were highly correlated with the content of the anthocyanin.

**Conclusions:**

The composition and contents of anthocyanins in green apple in UV-B-treatment very greatly. A series of metabolites and candidate genes were revealed through combined analysis of metabolome and transcriptome. These results provide an important data for dissecting candidate genes and molecular basis governing green apple color formation in response to visible light and UV-B light.

**Supplementary Information:**

The online version contains supplementary material available at 10.1186/s12870-021-03121-3.

## Background

In recent years, red apple have attracted the attention of consumers due to their attractive appearance and their reported nutritional value [1, 2]. Red apple varieties such as ‘Fuji’ [3] and ‘Fortune’ [4] can synthesize high numbers of anthocyanins and are preferred by more consumers. Conversely, green apple cannot synthesize anthocyanins during natural growth, so that they mainly exhibit green or yellow colors, for example, the ‘white winter pearmain’ [5, 6] and ‘mutsu’ [4] apple varieties.

Annually, during apple growing seasons, particularly in the later stages of growth, sunlight plays important role in the development of fruit color [7–9]. In China, paper bagging is a tool applied in apple or pear fruit production [10–12], can not only control insect and bird pests, but can also enhance red pigmentation in the red fruit varieties in the late stages of fruit growth [7]. In addition, the degree of red pigmentation in one fruit variety could vary significantly in one location across several years based on varying sunlight intensity and temperature in an environment. Early studies suggested that light and ultraviolet-B (UV-B) radiation regulate anthocyanin synthesis by regulating the activity of key enzymes and the gene transcription levels in anthocyanin biosynthesis pathway in red skin color fruit [13–15]. Furthermore, lowing the temperature is more beneficial to anthocyanin synthesis compared to high temperature in fruit peel [15]. Some researchers have attempted to elucidate the mechanisms of fruit coloration using high-throughput techniques [7, 9, 13]. However, to date, the effect of visible light and UV-B on coloration of red skin color and green skin color apple fruit, and their molecular foundation is rarely studied.

The red coloration in red apple varieties is due to the synthesis of anthocyanin in exocarp [4, 15]. Anthocyanins are synthesized through flavonoid biosynthesis pathways, and the biosynthesis of anthocyanins involves several key enzymes and the genes expressions of coding these enzymes, it mainly contains phenylalanine ammonia-lyase (PAL), chalcone synthase (CHS), chalcone isomerase (CHI), flavanone-3-hydroxylase (F3H), dihydroflavonol 4-reductase (DFR), anthocyanidin synthase (ANS), and UDP-glucose: flavonoid 3-glucosyltransferase (UFGT) [16]. Regulating the genes encoding these enzymes are regulated by a protein complex formed by an R2R3-MYB, a basic-helix-loop-helix, and a WD40 [17]. The complex induces gene expression by binding to the promoters of structural gene [18]. In numerous species, MYB transcription factors (TFs) are considered essential for the activation of anthocyanin biosynthesis related genes. In apple, *MdMYB1* and *MdMYB10* act as positive TFs for anthocyanin biosynthesis in fruit skin [19, 20], while *MdbHLH3* plays a regulatory role during anthocyanins biosynthesis [21]. Although the research on apple fruit coloring mainly focuses on red varieties, but few people have studied green varieties, particularly, the response of green apple varieties to visible light and UV-B exposure. Therefore, it is critical to investigate the potential mechanisms by which visible light and UV-B induce anthocyanin synthesis in green apple varieties.

In the present study, we reported the anthocyanin accumulation by metabolome and transcriptome analyses in ‘white winter pearmain’ apple fruit peel samples treated with artificial visible light (63 h), artificial UV-B radiation (63 h), and a control (CK, bagged fruit stored in the dark for 63 h) after harvesting. Based on an integrated analysis of the differential metabolites and gene expression levels, we identified the metabolites involved in visible light and UV-B-responsive reactions, and analyzed the differential regulation of structural genes involved in anthocyanins biosynthesis. The result of the present study could elucidate further the mechanisms by which visible light and UV-B regulate anthocyanin biosynthesis in apple. In addition, the metabolomic and transcriptomic data obtained would offer basic data that would facilitate further investigations on the influence of visible light- and UV-B-induced fruit coloration in green apple varieties.

## Results

### Changes in fruit pigmentation patterns in the peel of ‘white winter pearmain’ fruit

**Fig. 1:**
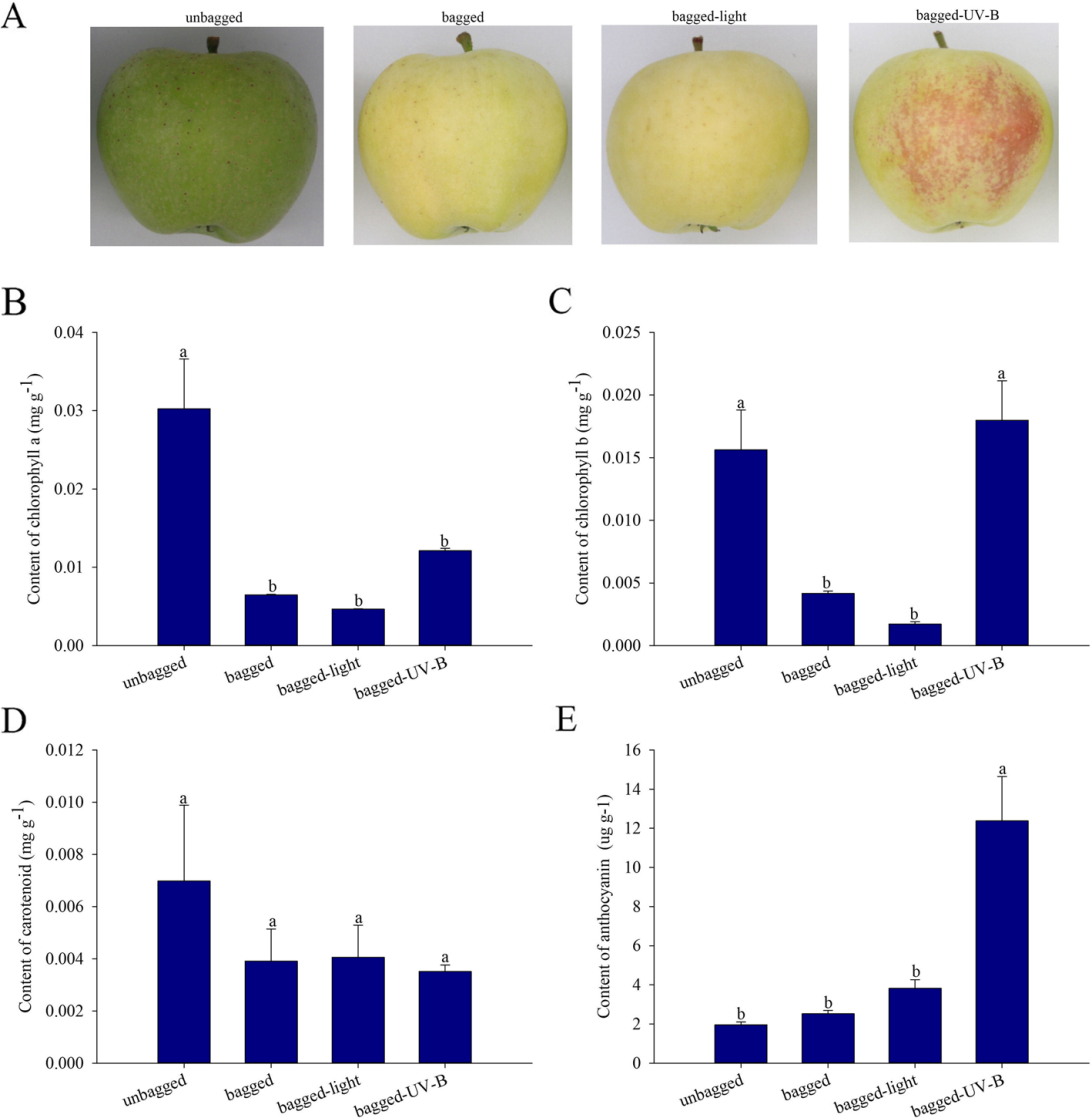
Changes in pigmentation in fruit peel of ‘white winter pearmain’ in unbagged, bagged, bagged-light-, and bagged-UV-B-treated samples (63 h). **A** Color changes in ‘white winter pearmain’ during treatment. Changes in concentrations of chlorophyll a (**B**), chlorophyll b (**C**), carotenoids (**D**), and anthocyanins (**E**) during treatment. The graphs show average values from six independently sampled fruit as biological replicates. Error bars are the standard deviation. *P* < 0.05

Following transportation to the laboratory and upon visual inspection, the unbagged ‘white winter pearmain’ fruit were green and the bagged fruit were pale yellow. In addition, the fruit removed from the bags and subjected to light treatment were pale yellow after 63 h, while the fruit removed from the bags and subjected to UV-B radiation treatment were red after 63 h (Fig. 1A). The red coloration was accompanied by an increase of anthocyanin concentration. In addition, the contents of carotenoid, chlorophyll a and chlorophyll b in the bagged and light-treatment fruit were very low, while the chlorophyll b and anthocyanin accumulation levels increased significantly following treatment with UV-B radiation (Fig. 1B-E).

### Metabolic differences among the light, UV-B, and CK peel treatments

**Fig. 2:**
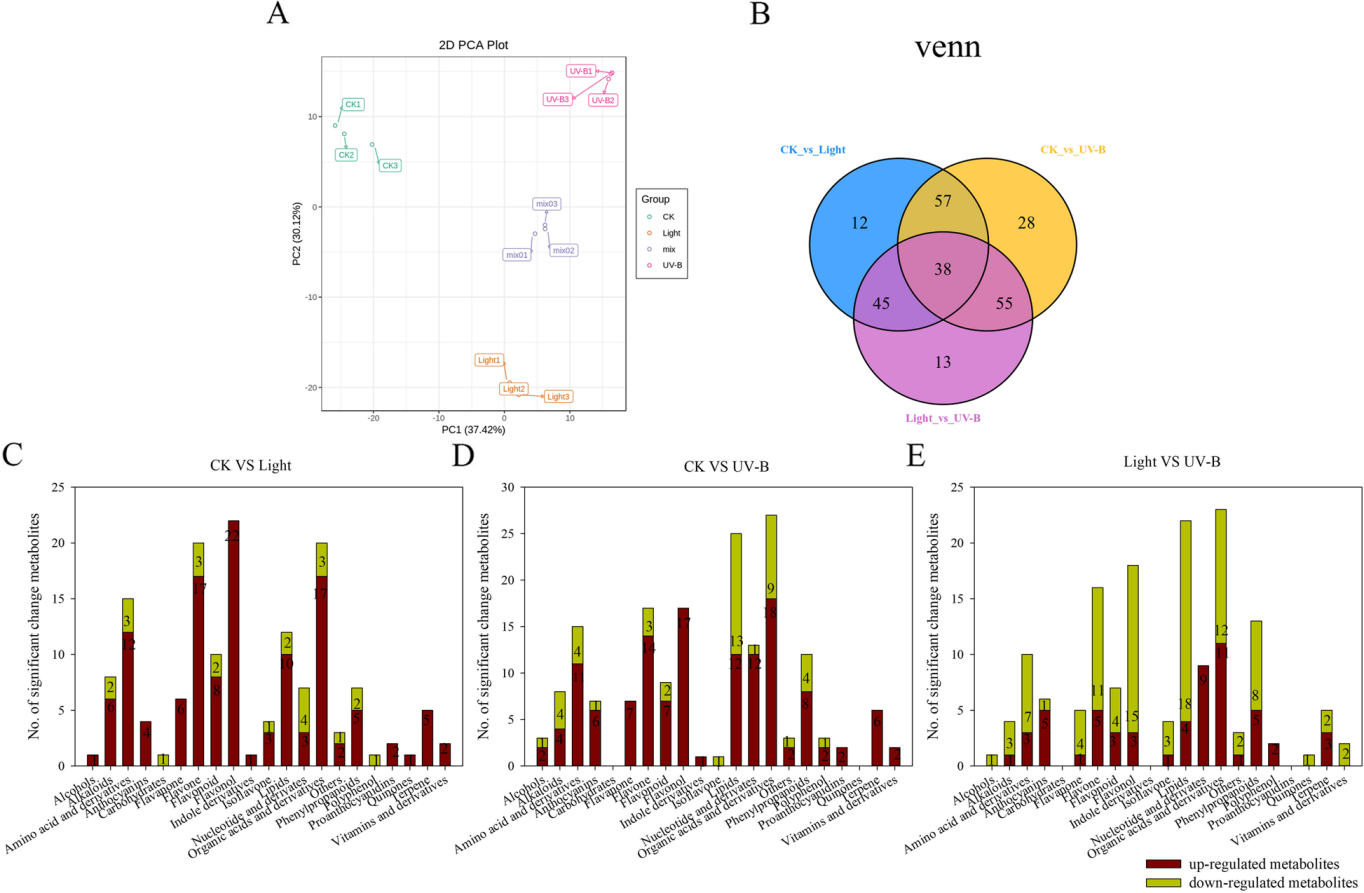
Significantly changed metabolites (SCMs) in visible light and UV-B radiation treated fruit compared to in the metabolites in CK. **A** Principal components analysis score-plot of metabolite profiles from the light, UV-B, and CK treatments. **B** Venn plot of metabolite profiles in the light, UV-B, and CK treatments. **C**-**E** Number of SCMs under the dark (CK), visible light and UV-B radiation treatments

To compare the differences of metabolite compositions among the light, UV-B, and CK peel treatments, the peel sampled were subjected to LC–MS/MS analysis. In the present study, among the 248 metabolites, there were 58 flavonoids and 8 anthocyanins, a major phenylpropane metabolic pathway, were identified and quantified in apple peel (Table S2). Principal components analysis (PCA) revealed that the CK, light, and UV-B treatments were clearly separated in the PC1–PC2 score plots (Fig. 2A). In addition, a Venn diagram of the dataset revealed that 210 metabolites were expressed differentially between the CK and light treatments, between the CK and the UV-B radiation treatment, as well as between the light and the UV-B radiation treatment (Fig. 2B). Among the 248 metabolites identified in the apple peel, 152 and 178 (61.3 and 71.8%) changed substantially in the light and the UV-B treatments, respectively, compared to in the CK treatment. In addition, 127 and 133 metabolites were upregulated, and 25 and 45 metabolites were downregulated in the light and UV-B treatments, respectively, compared to the metabolites in the CK treatment (Fig. 2C-D). Moreover, 57 metabolites were upregulated and 94 metabolites were downregulated in the UV-B treatment compared with the light treatment (Fig. 2E).

### Analysis of metabolic pathways and metabolites under the light- and UV-B-treatment

**Fig. 3:**
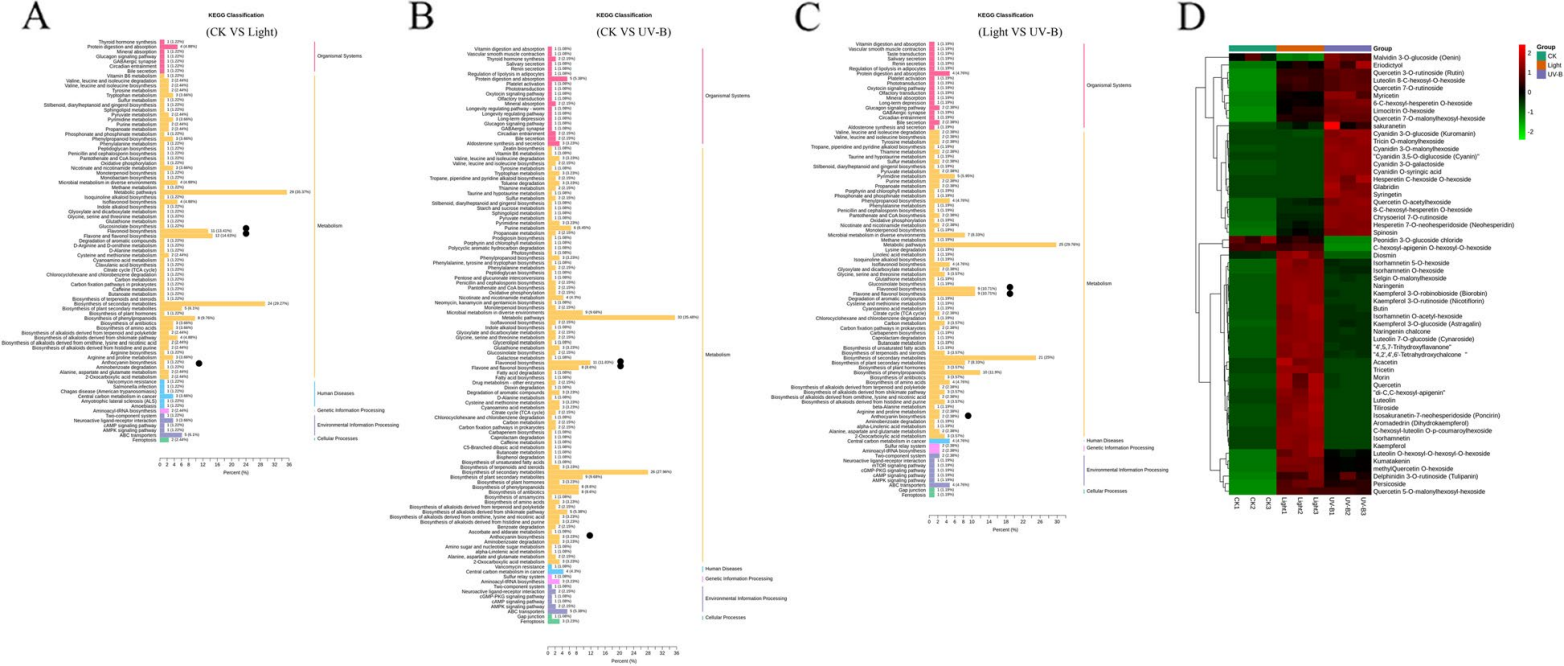
KEGG pathway enrichment of metabolites under the dark (CK), visible light and UV-B radiation treatments. **A**-**C** KEGG pathway enrichment of metabolites under the dark (CK), visible light and UV-B radiation treatments. **D** Heat map of 58 selected metabolites. The phenylpropanoid pathway metabolites were the most significantly changed metabolites. Black dot indicates flavonoid, flavone and flavonol, and anthocyanin biosynthesis metabolism

To identify different metabolites in the peel of light, UV-B compared to those of CK, UV-B compared to those of light, a metabolom comparison of the materials mentioned above was carried out. A KEGG pathway enrichment analysis revealed that the significantly changed metabolites were enriched mainly in ‘phenylpropanoid metabolic processes’ including flavonoid biosynthesis, flavanone biosynthesis, flavone biosynthesis, flavonol biosynthesis, and anthocyanin biosynthesis (Fig. 3A-C). Fifty flavonoids and 8 anthocyanins with significant changes in the metabolites of flavonoid and astaxanthin were screened out from the metabolome data for heat map analysis (Fig. 3D, Table S3). The results showed that the compounds of related to flavonoids and anthocyanins increased significantly under visible light and UV-B induction, such as cyanidin 3,5-O-diglucoside, cyanidin 3-O-galactoside, delphinidin 3-O-rutinoside, quercetin and so on.

### Differential expression of genes in the fruit peel

**Fig. 4:**
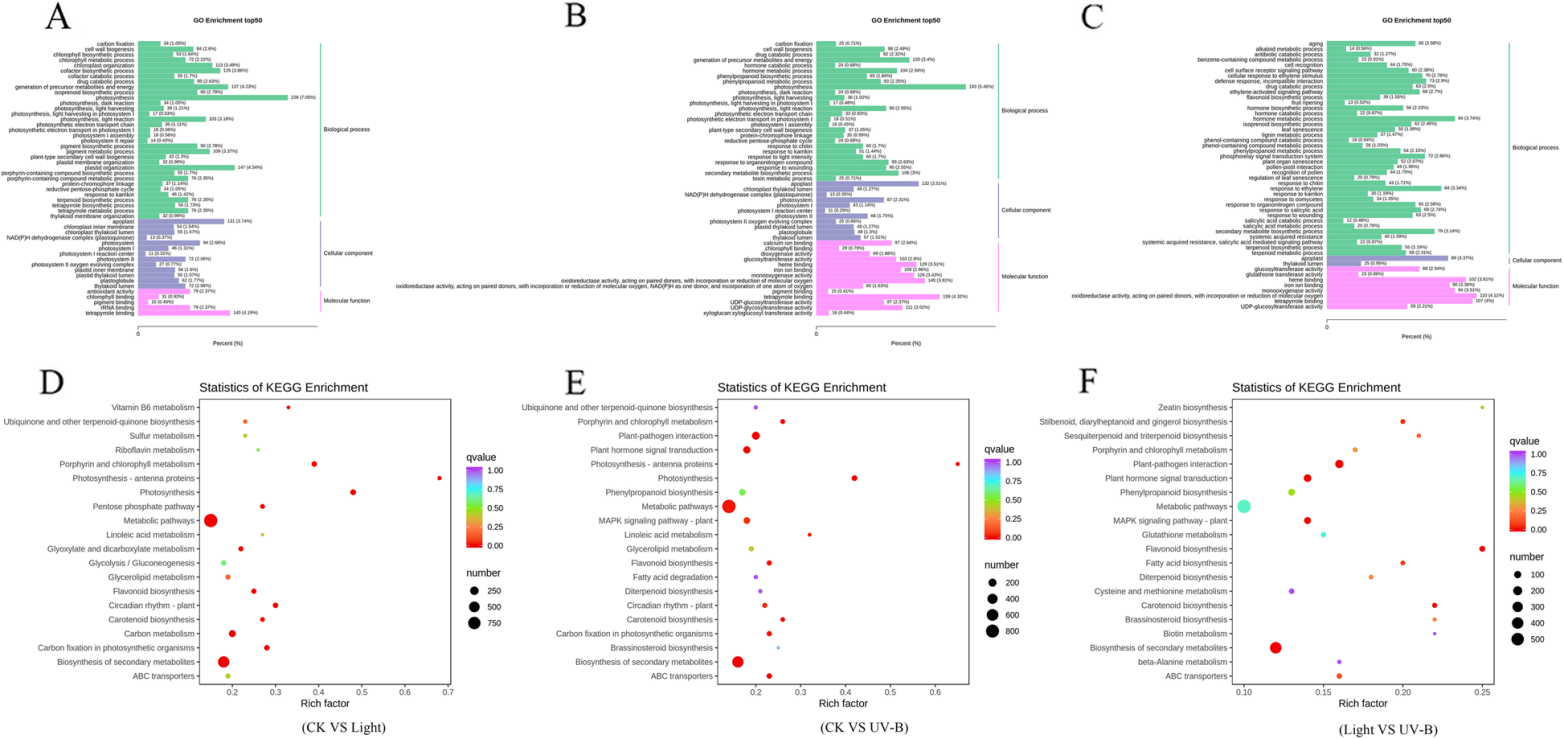
GO classification and KEGG pathway enrichment analyses of differentially expressed genes (DEGs) under the dark (CK), visible light and UV-B radiation treatments. **A**-**C** GO functional classification of DEGs. GO enrichment of DEGs, which are associated with photosynthesis and UDP-glycosyltransferase activity. **D**-**F** Top 20 KEGG pathway enriched DEGs. The significantly changed metabolites in the metabolic pathway include those involved in flavonoid biosynthesis. Number of DEGs is represented by the size of the circle

To identify the differentially expressed genes (DEGs) among the light, UV-B, and CK treatments, a transcriptomic comparison was performed. PCA score and Venn plots of gene expression among treatments revealed the effectiveness of data (Figure S1). Based on fold change ≥ 2 and FDR < 0.05 criteria, 37,110 and 37,709 DEGs were identified in the light and UV-B treatments compared with CK, respectively, and 3469 and 4212 genes, respectively, were significantly upregulated (Table S4a-b). In addition, 37,590 DEGs were identified in the UV-B compared with light, and 2762 genes were significantly upregulated (Table S4c). GO enrichment top 50 analysis of the 3355, 3428 and 2659 DEGs showed that the three major biological processes were biological process, cellular component, and molecular function. In the molecular function domain, the DEGs were enriched in catalytic activity and binding. In the cellular component domain, most of the DEGs were enriched in four categories, including apoplast, chloroplast, plastid, and thylakoid lumen (Fig. 4A-C). KEGG pathway enrichment analysis revealed that the DEGs were mainly enriched in various metabolic processes including flavonoid biosynthesis and secondary metabolism biosynthesis (Fig. 4D-F).

### Changes of flavonoid and anthocyanin biosynthesis

**Fig. 5:**
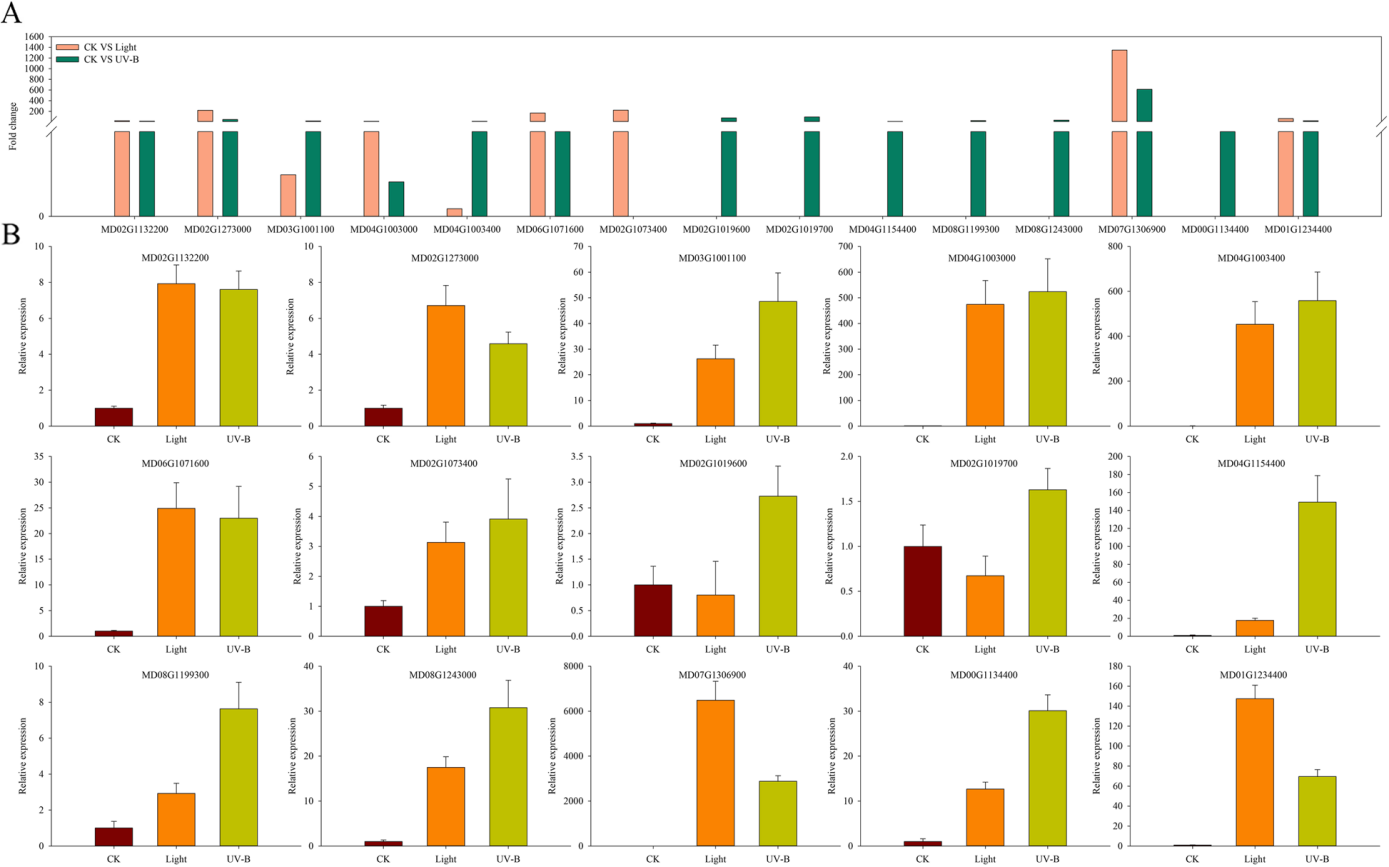
Expression levels of differentially expressed genes (DEGs) in the flavonoid and anthocyanin pathways under the visible light and UV-B radiation treatments compared to the expression levels in the CK treatment. **A** Comparison of flavonoid and anthocyanin associated mRNA expression levels detected by RNA-seq. **B** qRT-PCR analysis results showing the expression levels of 15 flavonoid and anthocyanin-associated DEGs under the dark (CK), visible light and UV-B radiation treatments

Transcriptomic data revealed that flavonoid and anthocyanin biosynthesis genes were differentially induced by light and UV-B radiation treatments (Fig. 5A). We selected 15 significantly different expressed genes to validate the results of the transcriptomic analysis. qRT-PCR results revealed that 10 out of the 15 genes were expressed at higher levels under UV-B than under the light treatment. Particularly, the level of expression of *MD00G1134400*, a *UFGT* homologue, was enhanced under the UV-B treatment, implying the requirement of such structural enzymes in anthocyanin biosynthesis in apple (Fig. 5B).

### Integrated analyses of the metabolomic and transcriptomic datasets

**Fig. 6:**
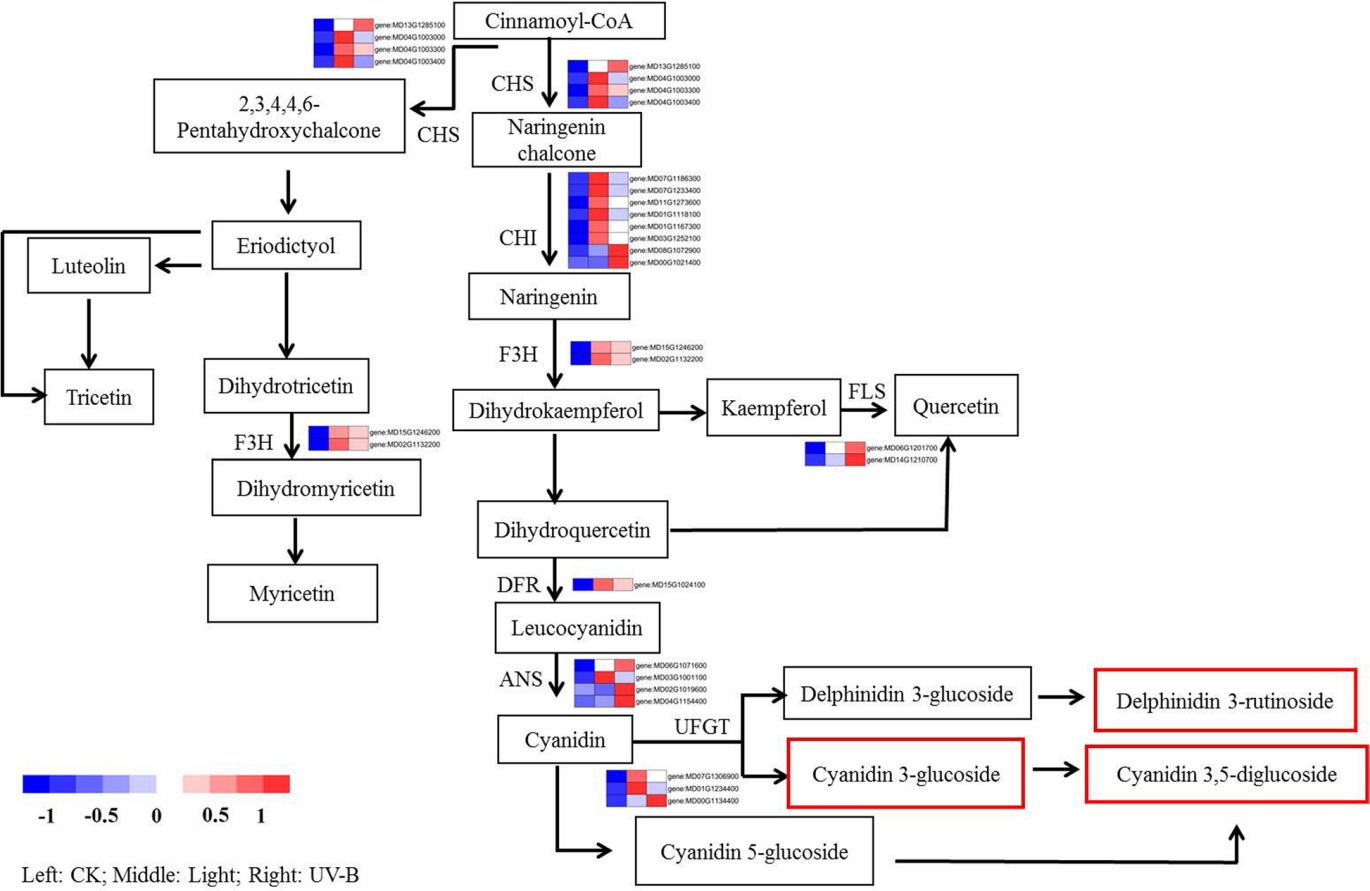
Metabolites and genes associated with the flavonoid and anthocyanin biosynthesis pathways were analyzed using metabolomic and transcriptomic approaches. Heat maps of differentially expressed genes (DEGs) in transcripts in flavonoid and anthocyanin biosynthesis. CHS, chalcone synthase; CHI, chalcone isomerase; F3H, naringenin 3-dioxygenase; FLS, flavonoid 3’-monooxygenase; DFR, dihydroflavonol 4-reductase; ANS, anthocyanidin synthase; UFGT, UDP glucose-flavonoid 3–0-glucosyltransferase. Differential metabolites associated with anthocyanin synthesis are represented in the red box under the dark (CK), visible light and UV-B radiation treatments

To reveal the regulatory mechanisms of enhanced flavonoid and anthocyanin biosynthesis in peel exposed to light and UV-B radiation, 58 metabolites (flavonoid biosynthesis, 50; and anthocyanin metabolism, 8) and 30 DEGs (flavonoid biosynthesis, 27; and anthocyanin metabolism, 3) were selected for further analyses (Fig. 6). Although a vast number of flavonoids were detected in the peel subjected to light and UV-B radiation treatments (Table S3), UV-B radiation induced higher anthocyanin concentrations than the light treatment (Fig. 1A). Particularly, the compounds that demonstrated changes, including cyanidin 3, 5-O-diglucoside (cyanin) and cyanidin 3-O-glucoside (kuromanin), showed markedly higher abundances under the UV-B than under the visible light treatment (Table S3). In addition, we arranged 30 DEGs based on their corresponding positions in the apple flavonoid biosynthesis pathway (Fig. 6). Among them, some *MdCHS*, *MdCHI*, *MdF3H*, *MdFLS*, *MdDFR*, *MdANS*, and *MdUFGT* homologues exhibited different expression levels.

### Differential expression of anthocyanin biosynthetic genes in fruit peel

**Fig. 7:**
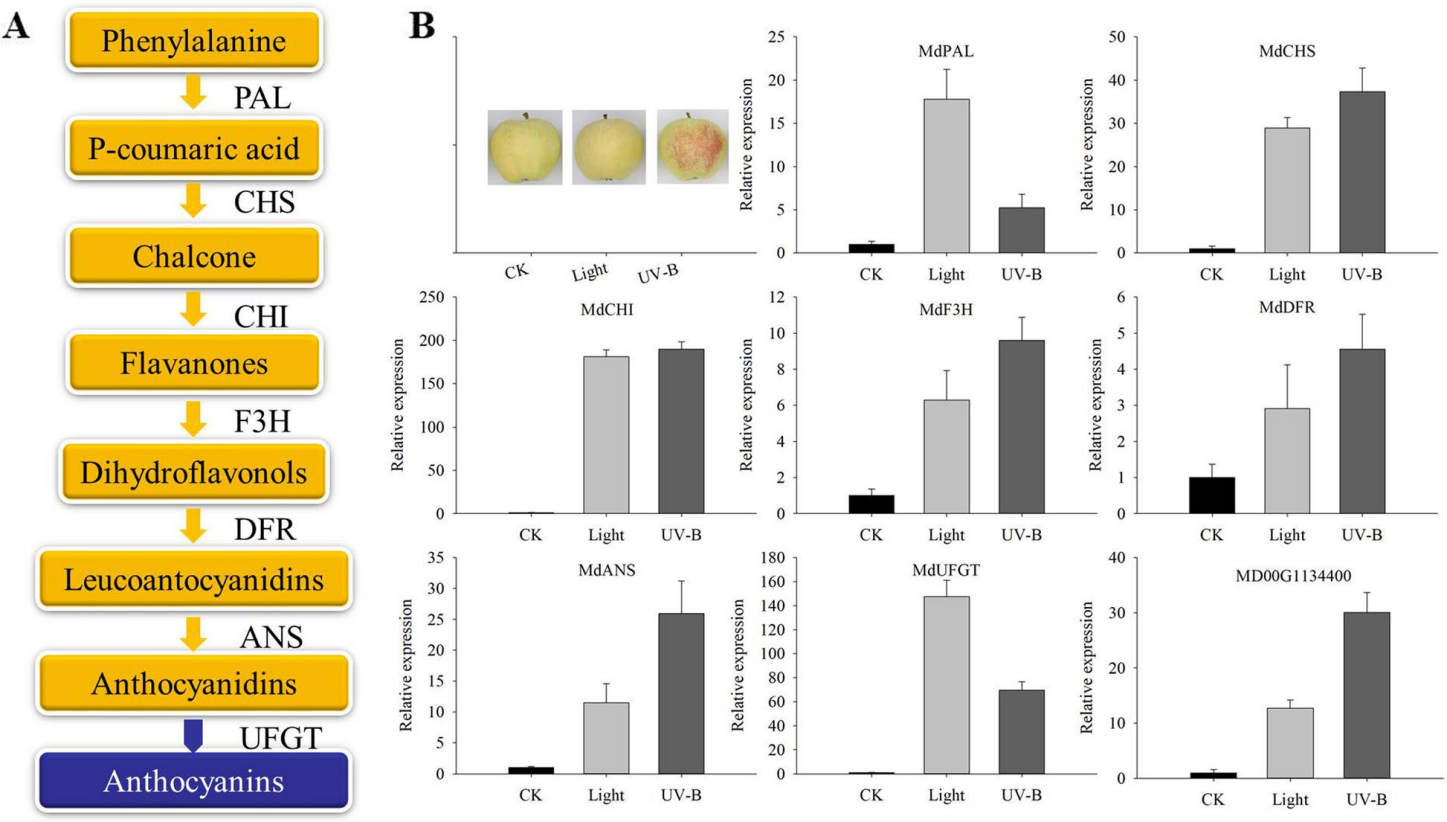
Biosynthetic pathways of anthocyanins in apple. **A** The key genes of the anthocyanin biosynthesis pathway. **B** qRT-PCR results showing the expression levels of 8 anthocyanin-related genes under the dark (CK), visible light and UV-B radiation treatments in the peel. PAL, phenylalanine ammonia-lyase, XM008357397; CHS, chalcone synthase, XM029091251; CHI, chalcone isomerase, XM008394013; F3H, naringenin 3-dioxygenase, AB074486; DFR, dihydroflavonol 4-reductase, AY227728; ANS, anthocyanidin synthase, AB074487; UFGT, UDP glucose-flavonoid 3–0-glucosyltransferase, MD01G1234400

Anthocyanin in the skins investigated was biosynthesized via the flavonoid pathway (Fig. 7A). To determine the roles of anthocyanin biosynthetic genes under the light and UV-B conditions, the expression patterns of genes involved in their biosynthesis were analyzed. Significant expression changes were observed in both the light and UV-B responsive genes. Pearson’s correlation analysis showed that the genes expression levels in transcriptome verification experimrnt was significantly correlated with the reported transcription levels of anthocyanin biosynthesis related genes (*P* < 0.05, and *P* < 0.01) by light and UV-B treatment in apple peel (Table 1). We further performed qRT-PCR analysis on the reported related genes and candidate genes. The results showed that most of the genes, such as *MdCHS*, *MdF3H*, *MdDFR*, *MdANS*, and *Md00G1134400* were more responsive to UV-B induction (Fig. 7B). The results suggested that the contribution of UV-B induced anthocyanin synthesis may be greater than that of visible light in green apple.

**Table 1:** Pearson correlation coefficients between flavonoids and anthocyanin-related genes in transcriptome and the reported genes of related to anthocyanin biosynthesis under light and UV-B treatment in apple peel

	MD02G1132200	MD02G1273000	MD03G1001100	MD04G1003000	MD04G1003400	MD06G1071600	MD02G1073400	MD02G1019600	MD02G1019700	MD04G1154400	MD08G1199300	MD08G1243000	MD07G1306900	MD00G1134400	MD01G1234400	MdPAL	MdCHS	MdCHI	MdF3H	MdDFR	MdANS
MD02G1132200	1																				
MD02G1273000	0.944	1																			
MD03G1001100	0.863	0.647	1																		
MD04G1003000	0.992	0.894	0.92	1																	
MD04G1003400	0.976	0.849	0.952	0.996	1																
MD06G1071600	1^*^	0.953	0.847	0.988	0.969	1															
MD02G1073400	0.955	0.802	0.974	0.985	0.997	0.945	1														
MD02G1019600	0.379	0.052	0.795	0.493	0.572	0.351	0.638	1													
MD02G1019700	0.139	-0.196	0.621	0.263	0.351	0.109	0.428	0.969	1												
MD04G1154400	0.553	0.246	0.898	0.654	0.721	0.527	0.776	0.981	0.902	1											
MD08G1199300	0.695	0.481	0.963	0.78	0.835	0.673	0.878	0.929	0.809	0.983	1										
MD08G1243000	0.877	0.668	1^*^	0.93	0.96	0.861	0.98	0.778	0.598	0.885	0.955	1									
MD07G1306900	0.854	0.978	0.474	0.782	0.721	0.87	0.661	-0.157	-0.396	0.039	0.22	0.499	1								
MD00G1134400	0.779	0.528	0.989	0.852	0.897	0.759	0.93	0.876	0.729	0.953	0.992	0.985	0.339	1							
MD01G1234400	0.868	0.983	0.498	0.798	0.739	0.883	0.681	-0.13	-0.371	0.066	0.246	0.522	1^*^	0.365	1						
MdPAL	0.724	0.911	0.276	0.631	0.556	0.745	0.486	-0.364	-0.583	-0.175	0.007	0.303	0.977	0.131	0.971	1					
MdCHS	0.965	0.825	0.965	0.991	0.999^*^	0.957	0.999^*^	0.607	0.392	0.751	0.858	0.972	0.689	0.915	0.709	0.519	1				
MdCHI	0.997	0.913	0.902	0.999^*^	0.991	0.994	0.976	0.454	0.22	0.619	0.752	0.913	0.808	0.828	0.824	0.665	0.984	1			
MdF3H	0.908	0.719	0.995	0.954	0.997	0.895	0.992	0.732	0.541	0.851	0.932	0.998^*^	0.558	0.97	0.581	0.368	0.986	0.94	1		
MdDFR	0.866	0.651	1^**^	0.922	0.954	0.85	0.975	0.792	0.616	0.896	0.962	1^*^	0.479	0.988	0.503	0.281	0.966	0.904	0.996	1	
MdANS	0.793	0.547	0.992	0.864	0.906	0.774	0.938	0.865	0.714	0.946	0.989	0.988	0.361	1^*^	0.386	0.154	0.924	0.84	0.975	0.991	1

* and ** significant at *P* <0.05 and 0.01 respecrivly

### Transcription factors (TF) and light signal transduction related genes of light-, UV-B-responsive

**Fig. 8:**
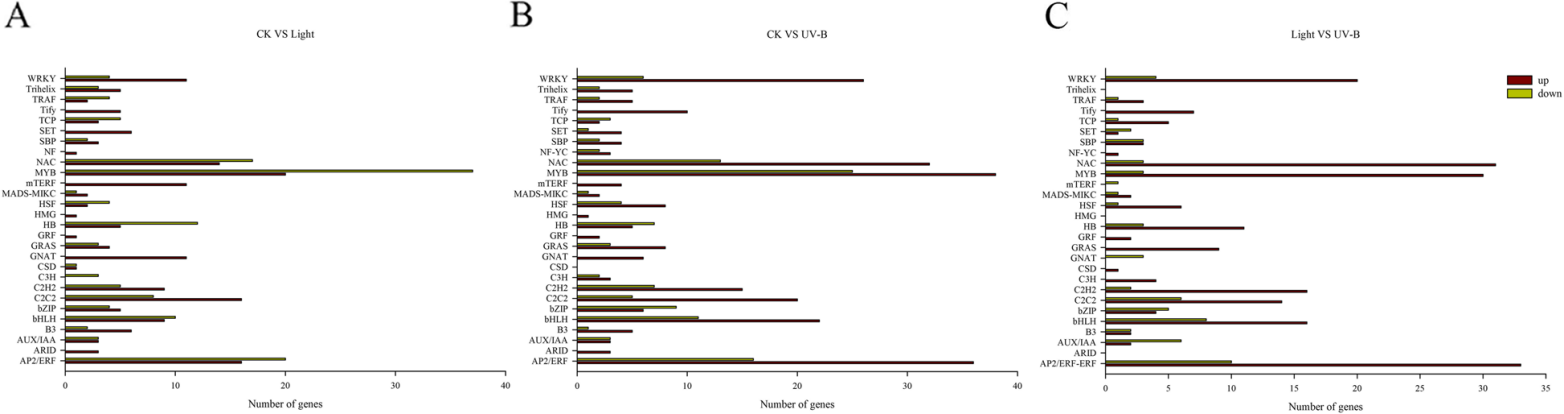
Transcription factor analysis under the dark (CK), visible light and UV-B radiation treatments in apple peel. The number of upregulated (red) and downregulated (green) transcription factor (TFs) are indicated

Three hundred eighty-two differentially expressed transcription factors belonging to 52 TF families, and 475 differentially expressed transcription factors belonging to 59 TF families were identified in the light-treatment and the UV-B treatment compared with CK, respectively, and 346 differentially expressed transcription factors belonging to 48 TF families were identified in the UV-B treatment compared with light-treatment (Table S5). In addition, we observed the up-regulation and down-regulation of 28 TF families that were both regulated by light and UV-B radiation treatments, including WRKY, NAC, MYB, bHLH, and AP2/ERF families (Fig. 8). Although rather similar numbers of TFs were observed between the light and the UV-B radiation treatments, most of the TFs were upregulated in UV-B. In the gene set, AP2/ERF was the largest family with 47 members, followed by the MYB, NAC, bHLH, and WRKY families, with 46, 45, 33, and 32 members in the UV-B treatment, respectively (Fig. 8B, Table S5). And the numbers of TFs in UV-B treatment were signifficant increased than light treatment (Fig. 8C). The results showed that UV-B may increasing the accumulation of anthocyanin through upregulation of TFs.

**Fig. 9:**
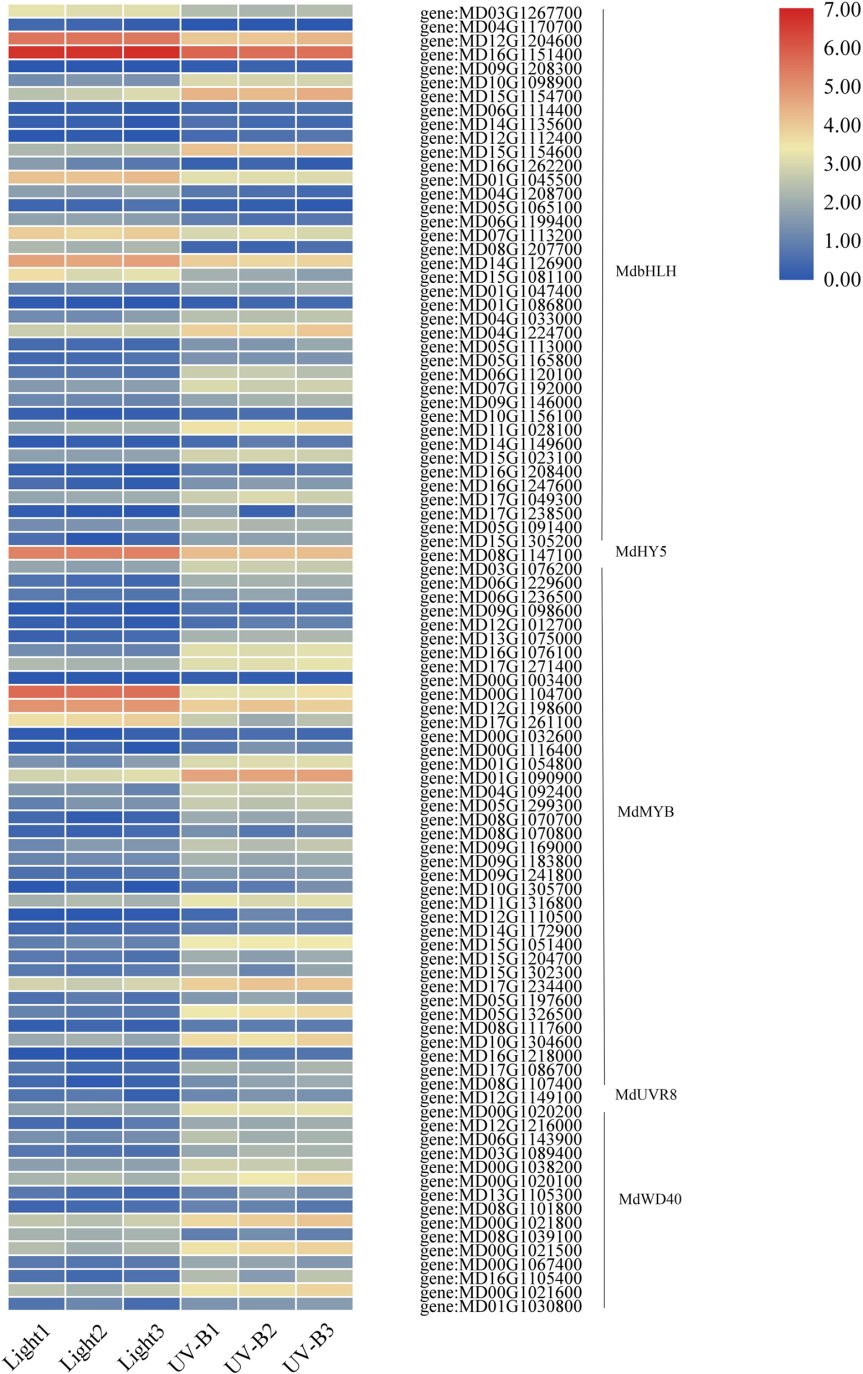
Heatmap representation of the expression patterns of light signal transduction related genes. Rows and columns in the heat map represent genes and samples collected. The color scale at the right represents the FPKM values. bHLH, basic helix–loop–helix; HY5, ELONGATED HYPOCOTYL; MYB, MYB family members; UVR8, UVB photoreceptor; WD40, WD40-repeat proteins

In addition, the transcriptional level of MYB, bHLH, and WD40 group genes were generally higher under UV-B treatment, and UV-B treatment showed tha same trend as the content anthocyanin. Here, we identified *MdHY5* (MD08G1147100) gene by transcriptom. The transcript level of *MdHY5* decreased under UV-B treatment in apple, which was poorly correlated with anthocyanin level. However, the transcript abundance of *MdUVR8* (MD12G1149100) was consistent with anthocyanin content, and UV-B induced the expression of *MdUVR8* was higher compared to light treatment in apple (Fig. 9).

## Discussion

### Coloration of bagged, unbagged, artificial light- and UV-B-treated apple fruit

Both light and UV-B radiation can induce coloration in red apple fruit [22, 23]. In nature, the red apple varieties can synthesize anthocyanins, which would appear red, and are influenced significantly by sunlight. Conversely, the green apple cannot synthesize anthocyanins and exhibits a green color even following exposure to sunlight [24]. Nevertheless, only a few studies have explored the responses of green apple fruit varieties to visible light and UV-B radiation. Previous studies have shown that bagging could facilitate fruit coloration [7]. Therefore, we bagged ‘white winter pearmain’ apple in the course of development. We observed that prolonged exposure to UV-B radiation could induce the coloration of green apple, while prolonged exposure to visible light had no effect (Fig. 1). The results indicated that green apple could synthesize red pigment under UV-B induction similar to red apple.

### Metabolites involved in the induction of coloration under visible light and UV-B radiation

Previous studies have reported that sunlight influences physiological change in fruit [11, 25]. In particular, sunlight influences the synthesis of phenols in late stages of fruit maturation [26], suggesting sunlight plays an important role in fruit development. There is evidence of an increase in anthocyanins and flavonoid compounds in fruit exposed to sunlight and that acclimation to sunlight could have positive effects on the growth of red apple varieties [9]. However, green fruit varieties do not exhibit responses in anthocyanin synthesis following exposure to sunlight [26]. In the present study, visible light and UV-B radiation could increase the accumulation of metabolites in fruit, mainly in the metabolic pathways of amino acids and their derivatives, as well as the metabolic pathways of anthocyanins, flavonoids (flavanones and flavonol), lipids, and organic acids, and their derivatives. In particular, the metabolites involved in the flavonoid metabolic pathway were up-regulated significantly (Fig. 2C, D). KEGG analysis showed that visible light and UV-B radiation induced metabolic and biosynthesis pathways, particularly flavonoid and anthocyanin biosynthesis pathways (Fig. 3A, B). In addition, visible light and UV-B radiation induced the synthesis of different flavonoids, and anthocyanins in 58 metabolites (Fig. 3D). The results indicated that there were significant differences in metabolite synthesis between visible light and UV-B radiation treatments. We further identified the metabolites associated with the anthocyanin biosynthesis pathway in the metabolome data, and observed that both visible light and UV-B radiation could induce the synthesis of delphinidin 3-rutinoside and cyanidin 3-glucoside, but only UV-B treatment could induce the formation of cyanidin 3, 5-diglucoside (Fig. 6, Table S3). Notably, cyanidin 3, 5-diglucoside is the dominant anthocyanin in rose flowers [27]. In the present study, cyanidin 3-glucoside and cyanidin 3, 5-diglucoside were the major anthocyanin components in the fruit treated with UV-B radiation. In addition, the concentrations of cyanidin 3, 5-diglucoside were 12.3-fold the concentrations of cyanidin 3-glucoside. Conversely, light treatment increased cyanidin 3-glucoside concentrations slightly and did not enhance cyanidin 3, 5-diglucoside production, which indicated that the red skin color could be attributed to the higher level of production of cyanidin 3, 5-diglucoside.

### Differentially expressed genes and other transcription factors are involved in fruit coloration

Sunlight facilitates the accumulation of different metabolic compounds by regulating plant gene expression [28]. Several studies have demonstrated that light induces anthocyanin synthesis through the expression of key enzyme genes in the anthocyanin biosynthesis pathway [16, 29]. In addition, numerous transcription factors associated with anthocyanin have been studied such as MdMYB1 and MdMYB10 [19, 20, 30, 31]. In the present study, Go and KEGG analyses revealed that the gene expression changes under visible light and UV-B radiation treatments were mainly in the metabolic pathways and the secondary metabolite pathways, particularly the flavonoid synthesis pathway (the results were consistent with the metabolome data) (Fig. 4). Transcriptome data showed that 6 out of the 15 genes involved in flavonoid biosynthesis were induced by both visible light and UV-B radiation, with one genes induced significantly by light, and the other five genes induced significantly by UV-B. In addition, three genes were closely associated with the anthocyanin biosynthesis pathway and were induced by both light and UV-B. GenBank analysis revealed that the three genes belonged to the same gene family, UFGT. At present, MD01G1234400 function (GenBank: AF117267) has been reported in several studies, and it is regulated by UV-B and temperature [15]. Our results showed that three genes, including MD01G1234400, MD07G1306900, and MD00G1134400, were induced by visible light and UV-B radiation. In particular, MD00G1134400 expression was induced more significantly by UV-B. qRT-PCR was used to verify gene expression, and the results were consistent with transcriptomic data (Fig. 5B).

We applied both metabolomic and transcriptomic techniques in the analysis of the effects of visible light and UV-B radiation on metabolites and genes expression in the flavonoid biosynthesis pathways. We identified 30 DEGs, including *MdCHS*, *MdCHI*, *MdF3H*, *MdFLS*, *MdDFR*, *MdANS*, and *MdUFGT* that responded to both visible light and UV-B radiation (Fig. 6). In addition, visible light and UV-B radiation induced the expression of TF family genes, mainly including MYB, bHLH, and WRKY. Particularly, the number and the transcription level of TFs induced by UV-B radiation was significant (Figs. 8 and 9). The results indicated that light and UV-B play important roles in the synthesis of phenylpropane, particularly in the flavonoid and anthocyanin synthesis pathways, and the effect of UV-B was apparent in the green apple fruit.

### Differential regulation of genes in anthocyanin biosynthesis

**Fig. 10:**
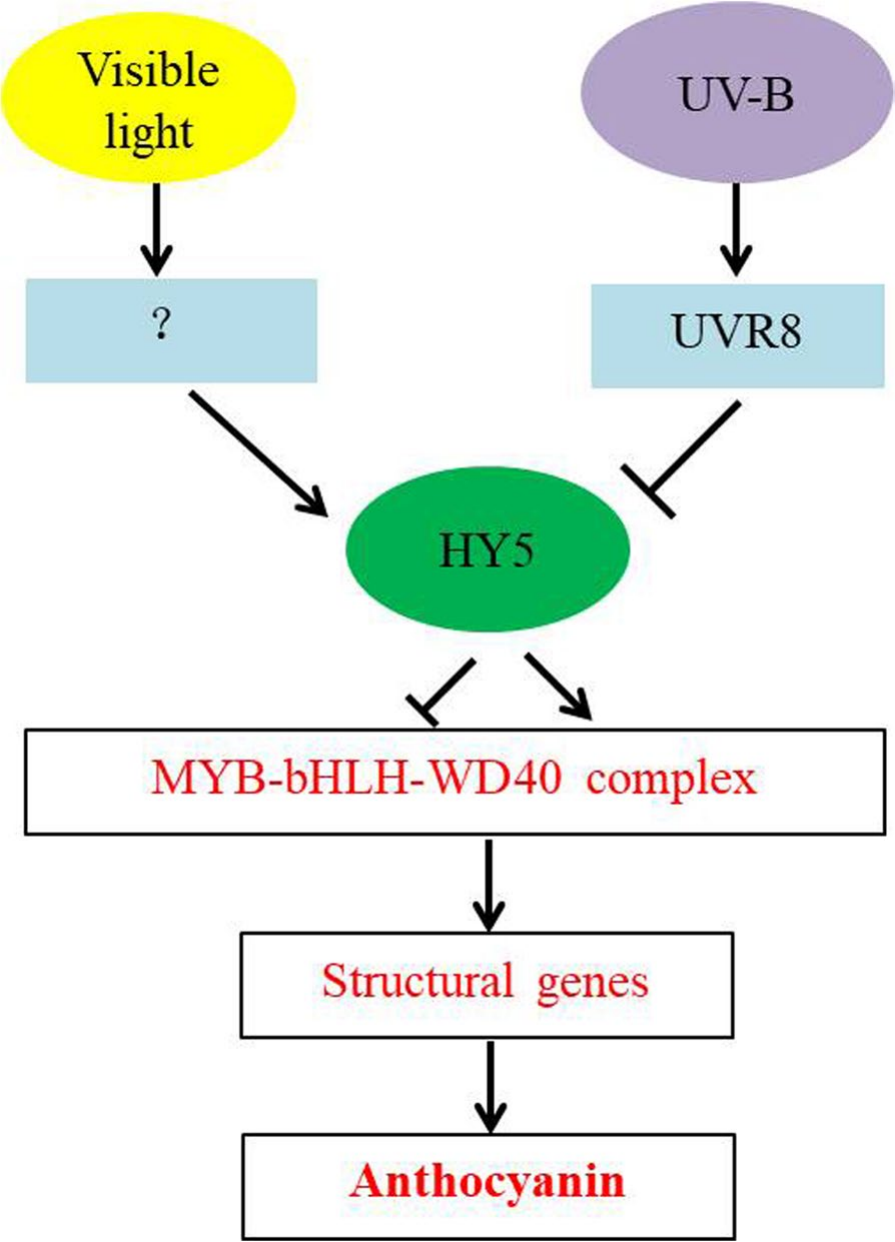
A proposed working hypothesis for light and UV-B-induced apple peel coloration. UVR8, UVB photoreceptor; HY5, ELONGATED HYPOCOTYL; MYB-bHLH-WD40, the complex of MYB, MYC-like basic helix–loop–helix (bHLH) and WD40-repeat proteins. ? represents visible light receptor has not been found under light-treatment

Anthocyanins are synthesized mainly via the flavonoid biosynthesis pathway [32]. In recent years, many studies have explored the key enzyme genes involved anthocyanin synthesis. Previous studies have reported that the anthocyanin synthesis pathway genes are regulated by sunlight in ‘Fuji’ apple [3, 9]. Ubi et al. [15] showed that the genes are induced strongly by low temperature. To verify that the key enzyme genes in the anthocyanin biosynthesis pathway are influenced by visible light and UV-B radiation in green apple, we analyzed the expression of associated genes. According to the results, both visible light and UV-B radiation could induce gene expression in the anthocyanin biosynthesis pathway. Particularly, UV-B radiation induced gene expression downstream significantly (Fig. 7). The results of the metabolomic and transcriptomic analyses revealed that UV-B induces the expression of anthocyanins such as cyanidin 3, 5-diglucoside in green apple, and the identification of the candidate genes involved requires further exploration. The correlation analysis confirmed that flavonoid and anthocyanin-associated genes in the transcriptome were closely related to the reported anthocyanin biosynthesis genes (Table 1). Therefore, it can be said that these genes were related to anthocyanin biosynthesis when apple are exposed to UV-B. In this study, green apple fruit were treated using visible light and UV-B radiation, and these results provide a theoretical basis that could facilitate the understanding of the effect of light and UV-B on the green fruit, and offer novel insights that could facilitate the mining of anthocyanin synthesis genes (Fig. 10).

## Conclusions

In this study, we analyzed the metabolome and transcriptome of ‘white winter pearmain’ apple fruit peel after harvesting and exposure to visible light and UV-B radiation. Using an integrated analysis of the differential metabolites and genes expression levels, we identified the metabolites involved in responses to visible light and UV-B, and further analyzed the differential expression of genes involved in anthocyanin biosynthesis using qRT-PCR. After data mining from transcriptome, a apple UDP glucose-flavonoid 3–0-glucosyltransferase gene, namely Md00G1134400, was found to be differentially expression of UV-B-responsive involved in anthocyanin biosynthesis in green apple fruit. In conclusion, the UV-B-induced red color of green apple may be caused by the regulation of UV-B-responsive signals, including UVR8 and members of the MBW complex, to regulate the transcriptional expression of structural genes of anthocyanin biosynthesis. So our results provide insights into the synthesis anthocyanin metabolic products and the basic gene-expression networks in green fruits exposed to UV-B radiation. Further work on gene function validation is required to have deep insights of the genetic and molecular mechanisms underlying green apple fruit coloration.

## Materials and methods

### Plant materials and experiments

The ‘white winter pearmain’ apple fruit were obtained from a commercial orchard in Taian, Shandong Province, China. In total, 150 fruitlets were covered with double layers of yellow-black paper bags 45 days after full blossom (DAFB). The fruit remained bagged before harvest (160 DAFB) and were transported to the laboratory with the remaining unbagged fruit. The fruit of artificial visible light-treatment (bagged fruit exposed to visible light [300–320 μmol m-2 s-1]) were placed approximately 10 cm (apple surface to visible light lamp), the wavelength of light was 400-700 nm, and the fruit of and artificial UV-B treatment (bagged fruit exposed to UV-B radiation [10–12 kJ m-2 s-1]) were placed approximately 25 cm (apple surface to UV-B lamp), the wavelength of UV-B lamp was 313 nm. The above two groups of treatments were performed on 60 fruit for 63 h after bag removal at room temperature (21 °C), respectively, and each treatment had three biological replicates, each replicate had 20 fruit. The control group (30 fruit) was included the bagged fruit maintained in the dark for 63 h. Fruit peel were scraped and immediately collected, quickly frozen in liquid nitrogen, and stored in a freezer at -80 °C. Three apple were used for each treatment, with three replicates. The phenotypes of the unbagged, bagged, bagged-light treatment, and bagged-UV-B treatment fruit were observed and recorded.

### Measurement of chlorophyll, carotenoid, and total anthocyanin concentrations

Pigments were extracted from fruit peel samples (0.3 g) using 3 ml 95% ethanol at room temperature (21 °C) in the dark until the peel tissues bleached completely according to previously described [33] with minor modification. The extracts were centrifuged at 8000 × *g* for 2 min, and the supernatants were collected and their absorbance was measured using a UV-1780 spectrophotometer (Shimadzu, Japan) at 665 nm, 649 nm, and 470 nm. The formulae for calculating chlorophyll concentrations were as following: chlorophyll a = 13.95*A665 – 6.88*A649; and chlorophyll b = 24.96*A649 – 7.32*A665. The formulae for calculating carotenoid concentrations were as following: carotenoids = (1000*A470 – 2.05*Ca – 114.8*Cb)/245 and the results were expressed as mg g^−1^.

Anthocyanin was extracted using 0.3 g of the frozen peel tissues in 3 ml 95% methanol:1.5 M hydrochloric acid (85:15) for 24 h at 4 °C in the dark according to previously described [34] with minor modifications. The extract was centrifuged at 12,000 × *g* for 5 min, and the supernatant was collected and analyzed using a UV-1780 spectrophotometer at 530, 620, and 650 nm. The anthocyanin concentrations were calculated using the following formula: 46,200*[(A530 -A620)-0.1*(A650-A620)] and the results were expressed as ug g^−1^.

### RNA-Seq analysis, cDNA synthesis, and data analysis

Total RNA was extracted from fruit peel of the CK, and the Light and UV-B treatment samples, and cDNA synthesis was performed using the RNA library prep kit (Tiangen, Beijing, China). At least three biological repeats were collected and mixed. The RNA-seq and data analysis were performed by Metware Biotechnology Co., Ltd. (Wuhan, China) as previously described [35–43] based on available genetic databases, including NCBI non-redundant protein database, the SwissProt database, the TrEMBL database, the Pfam database, the KOG database, the KEGG database, and the GO database, based on log2 fold changes > 1, and a false discovery rates (FDR) < 0.05. The experiments were repeated three times.

### Multiple reaction monitoring (MRM)

Multiple reaction monitoring (MRM) was performed by Metware Biotechnology Co., Ltd. (Wuhan, China). The freeze-dried apple peel were crushed using an MM 400 mixer mill (Retsch, Haan, Germany) for 1.5 min at 30 Hz. The powder (100 mg) was weighed and extracted overnight at 4 °C. After centrifugation at 10,000 × *g* for 10 min, the extracts were absorbed and filtered. The sample extracts were analyzed using a liquid chromatography coupled with tandem mass spectrometry (LC–MS/MS) system (HPLC, High Performance Liquid Chromatography, Shim-pack UFLC SHIMADZU CBM30A system, https://www.shimadzu.com.cn/; MS/MS, Tandem mass spectrometry, Applied Biosystems 6500 QTRAP, http://www.appliedbiosystems.com.cn/). The analytical conditions were as follows: HPLC: column, Waters ACQUITY UPLC HSS T3 C18 (1.8 μm, 2.1 mm × 100 mm); solvent system, water (0.04% acetic acid): acetonitrile (0.04% acetic acid); gradient program, 95:5 V/V at 0 min, 5:95 V/V at 11.0 min, 5:95 V/V at 12.0 min, 95:5 V/V at 12.1 min, 95:5 V/V at 15.0 min; flow rate, 0.4 mL/min; temperature, 40 °C; injection volume: 2 μl. The mass spectrum conditions mainly included an electrospray ionization (ESI) temperature of 500 °C, a mass spectrum voltage of 5500 V, and curtain gas pressure of 25 psi, with the collision-activated dissociation parameter set as high. In the triple quadrupole mass spectrometer, each ion pair was scanned according to the optimized declustering potential and collision energy [44]. A specific set of MRM transitions was monitored for each period according to the metabolites eluted within the period. The MRM was performed in triplicate for each treatment. The experiments were repeated three times.

### RNA extraction, DNA sequencing, and real-time quantitative reverse transcription (qRT-PCR) analysis

Samples stored in the freezer at -80 °C were used for total RNA extraction using a total RNA isolation system (Tiangen, Beijing, China). First-strand cDNAs were synthesized using a First-strand cDNA Synthesis Kit (Tiangen, Beijing, China). The full-length coding sequences of genes were isolated from ‘white winter pearmain’ cDNA. Real-time quantitative reverse transcription (qRT-PCR) analysis was performed according to MIQE guidelines [45, 46]. qRT-PCR was performed on a Bio-Rad CFX96TM Real-time PCR System using SYBR Real Master Mix (Transgen, Beijing, China) under the following PCR thermal cycling conditions: predenaturation at 95 °C for 30 s; followed by 39 cycles of 95 °C for 5 s, 60 °C for 15 s, and 72 °C for 20 s. MdActin (GenBank Accession Number: AB638619) was used as the housekeeping gene. The sequences of the primers used are listed in Supplementary Table S1. Three biological replicates were performed for each gene, and the standard curve method was applied in statistical analysis.

### Statistical analysis

Least significant differences (*p* < 0.05) were calculated for mean separation using Data Processing System procedures (Zhejiang University, Zhejiang, China). Standard errors were calculated and figures illustrated using Sigma Plot v12.0 (Systat Software Inc., San Jose, CA, USA). The data pearson correlation coefficients was analyzed using SPSS 16.0 software. For RNA-seq, differential expression analysis between two samples was performed using the DESeq2 package. After the differential expression analysis, the multiple hypothesis test correction of the P-value was performed using the Benjamini–Hochberg procedure based on a log2 fold change > 1 and an FDR < 0.05. The procedures were repeated three times.

## Supplementary Information

**Additional file 1: Table S1.** Primers for qRT-PCR.

**Additional file 2: Table S2.** a. Significantly changed metabolites in Light compared with CK. b. Significantly changed metabolites in UV-B compared with CK. c. Significantly changed metabolites in Light compared with UV-B.

**Additional file 3: Table S3.** Abundance of flavonoids and anthocyanins quantified by LC-MS/MS.

**Additional file 4: Table S4.** a-1. All differentially changed genes in Light compared with CK. a-2. Significantly differentially expressed genes in Light compared with CK. b-1. All differentially changed genes in UV-B compared with CK. b-2. Significantly differentially expressed genes in UV-B compared with CK. c-1. All differentially changed genes in UV-B compared with Light. c-2. Significantly differentially expressed genes in UV-B compared with Light.

**Additional file 5: Table S5.** a. Differentially expression transcription factors of Light compared with CK. b. Differentially expression transcription factors of UV-B compared with CK. c. Differentially expression transcription factors of Light compared with UV-B.

**Additional file 6: Figure S1.** Differentially expressed genes (DEGs) under the dark (CK), visible light and UV-B radiation treatments. (A) PCA score plot of genes profiles from the Light, UV-B, and CK. (B) Venn plot of genes profiles from the Light, UV-B, and CK.

## Data Availability

We have uploaded the RNA sequencing data to NCBI and the link is https://www.ncbi.nlm.nih.gov/bioproject/PRJNA623676. The data sets supporting the results of this article are include within the article and supplementary table.

## Abbreviations

UV-B: Ultraviolet-B
TFs: Transcription factors
qRT-PCR: Quantitative reverse transcription PCR
PAL: Phenylalanine ammonia-lyase
CHS: Chalcone synthase
CHI: Chalcone isomerase
F3H: Naringenin 3-dioxygenase
DFR: Dihydroflavonol 4-reductase
ANS: Anthocyanidin synthase
UFGT: UDP glucose-flavonoid 3–0-glucosyltransferase
DAFB: Days after full blossom
MRM: Multiple reaction monitoring
ESI: Electrospray ionization
PCA: Principal components analysis
DEGs: Differentially expressed genes
SCMs: Significantly changed metabolites
GO: Gene Ontology
KEGG: Kyoto Encyclopedia of Genes and Genomes
