# Supplementary material for: Metabolome and transcriptome profiling provide insights into green apple peel reveals light- and UV-B-responsive pathway in anthocyanins accumulation
Source: BMC Plant Biol. 2021 Jul 24;21:351. doi: 10.1186/s12870-021-03121-3 (PMC8305501; doi:10.1186/s12870-021-03121-3)
Supplement: Supplementary file 6 — Additional file 6: Figure S1. Differentially expressed genes (DEGs) under the dark (CK), visible light and UV-B radiation treatments. (A) PCA score plot of genes profiles from the Light, UV-B, and CK. (B) Venn plot of genes profiles from the Light, UV-B, and CK. [file 12870_2021_3121_MOESM6_ESM.doc]

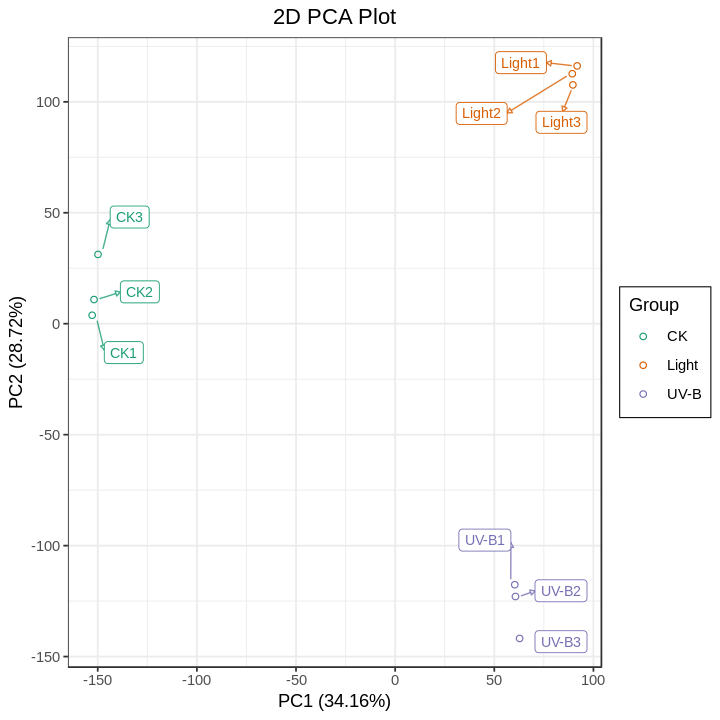

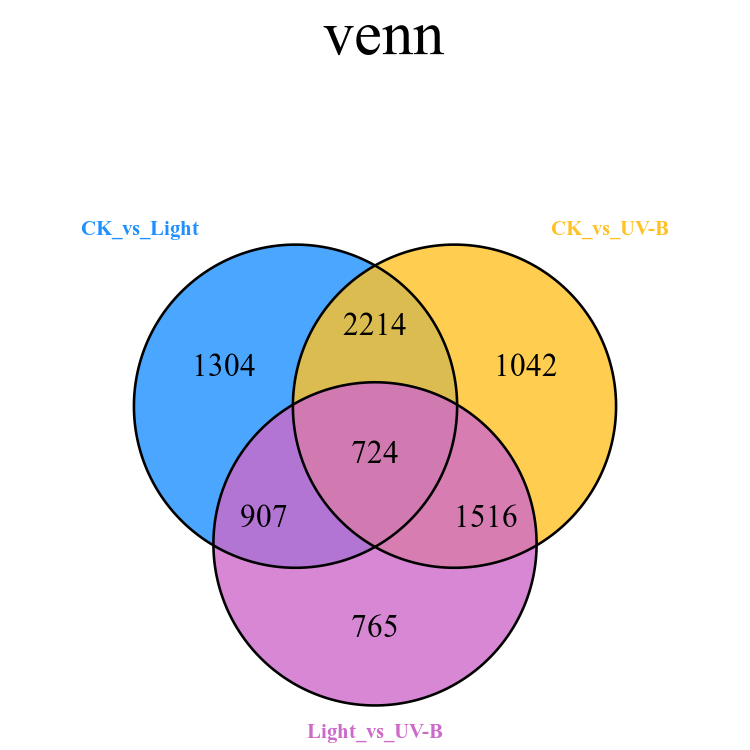


(A)

(B)

Supplemental **Figure.1 Differentially expressed genes (DEGs) under the dark (CK), visible light and UV-B radiation treatments.** (A) PCA score plot of genes profiles from the Light, UV-B, and CK. (B) Venn plot of genes profiles from the Light, UV-B, and CK.
